# Epiploic Appendagitis Unveiled: A Case of Misleading Abdominal Pain With Omental Fat Necrosis

**DOI:** 10.7759/cureus.62120

**Published:** 2024-06-10

**Authors:** Aqeel S Mahmood, Ahmed A Shakir, Mustafa Ismail

**Affiliations:** 1 Department of Surgery, College of Medicine, University of Baghdad, Baghdad, IRQ; 2 Department of Surgery, Baghdad Teaching Hospital, Baghdad, IRQ

**Keywords:** laparoscopic surgery, diagnostic imaging, acute abdominal pain, omental fat necrosis, epiploic appendagitis

## Abstract

Epiploic appendagitis is a benign inflammatory condition of the epiploic appendages, small fat-filled structures attached to the colon. Misdiagnosed frequently as more serious conditions like appendicitis or diverticulitis, it usually resolves with minimal treatment. This case report aims to emphasize the importance of recognizing epiploic appendagitis in differential diagnoses, highlighting the role of accurate imaging and surgical intervention in managing unusual presentations. We report a case involving a 27-year-old male who presented with acute, severe pain in the left iliac fossa. Initial assessments showed stable vital signs and negative virology screenings. Ultrasound imaging did not reveal any abnormalities in the abdominal organs but noted multiple gas-filled bowel loops and a 48 x 22 mm collection in the left iliac region. A CT scan with IV contrast further identified a 35 x 26 mm area of fat stranding in the left iliac fossa, indicative of epiploic appendagitis, and a 1 cm area of omental fat necrosis near the hepatic flexure. Persistent symptoms led to a diagnostic laparoscopy, which confirmed and treated gangrenous appendices epiploica. The patient’s postoperative recovery was uneventful, highlighting the effectiveness of surgical management. This case underscores the necessity for heightened awareness and diagnostic precision when encountering patients with acute abdominal pain that does not match common ailments. Early and accurate imaging, followed by timely surgical intervention if needed, can significantly improve outcomes by preventing complications from misdiagnosis or delayed treatment.

## Introduction

Epiploic appendagitis is a benign inflammatory condition of the epiploic appendages, small fat-filled pouches on the colon's exterior. It presents with symptoms akin to more severe abdominal emergencies, such as appendicitis and diverticulitis, leading to frequent misdiagnoses [1,2]. Computed tomography (CT) is the principal diagnostic tool, distinguishing epiploic appendagitis from other acute abdominal issues and guiding conservative management. This self-limiting condition often resolves with minimal treatment, such as non-steroidal anti-inflammatory drugs and, less commonly, antibiotics. The recognition of epiploic appendagitis is crucial to avoid unnecessary surgeries and reduce healthcare costs [3]. The rationale for this case report is to highlight the essential role of precise imaging in the diagnosis and management of epiploic appendagitis, aiming to improve treatment outcomes and patient care. This paper aims to present a case report involving a patient with acute abdominal symptoms and intraoperative peritoneal adhesions, discussing the diagnostic challenges and management decisions encountered. This case underscores the importance of considering epiploic appendagitis in differential diagnoses to ensure accurate assessment and appropriate treatment strategies.

## Case presentation

**Figure 1:**
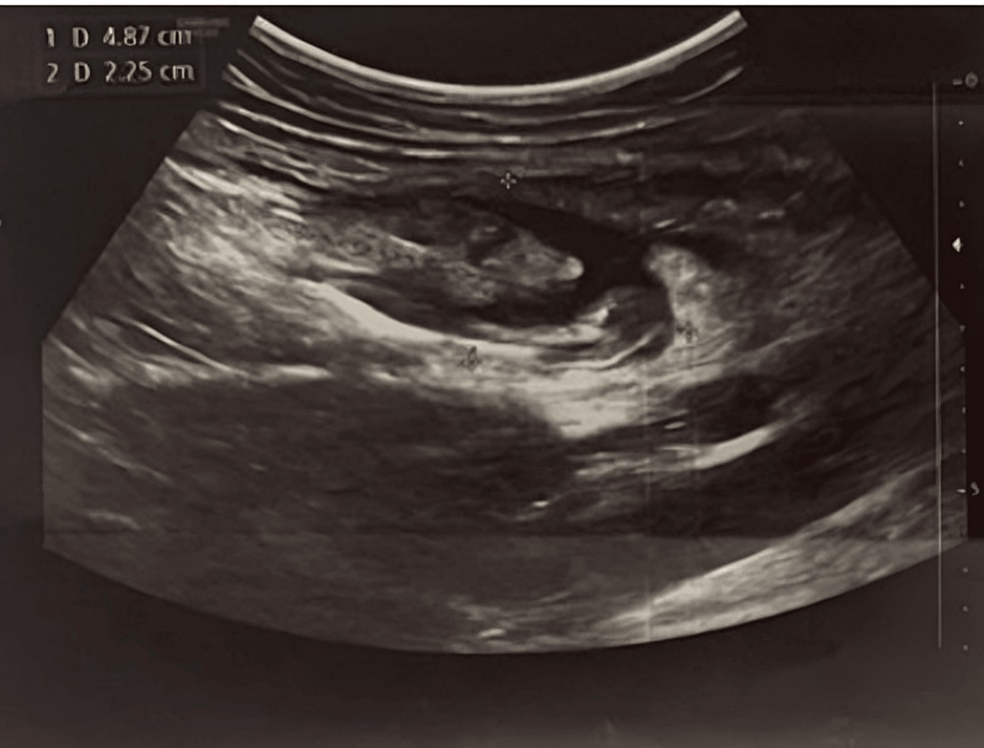
Preoperative abdominal ultrasound shows a collection measuring 48 x 22 mm in the left iliac region.

A 27-year-old male with a negative past medical history presented with acute severe pain in the left iliac fossa. Initial vital signs were stable with a blood pressure of 113/70 mmHg, a pulse rate of 90 bpm, and body mass index of 25. Abdominal ultrasonography revealed normal liver, gallbladder, kidneys, pancreas, spleen, and urinary bladder with no evidence of masses or stones. However, multiple gas-filled bowel loops and a collection measuring 48 x 22 mm in the left iliac region were noted (Figure 1). A subsequent CT scan with IV contrast confirmed fat stranding in the left iliac fossa measuring approximately 35 x 26 mm, consistent with epiploic appendagitis, along with a 1 cm area of omental fat necrosis near the hepatic flexure.

**Figure 2:**
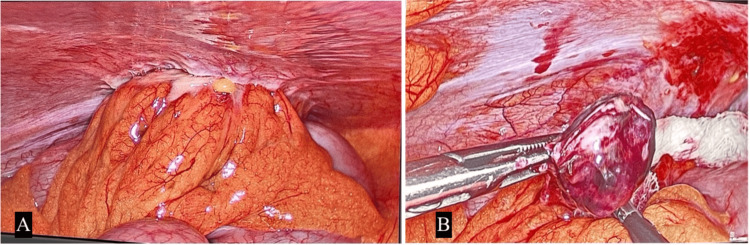
This intraoperative laparoscopic image shows a clear view of the internal abdominal anatomy, focusing on a section of intestines with visible vascular structures on the surface. A small, yellowish lesion or structure is visible near the center (A), possibly indicative of a pathological finding such as omental fat necrosis (gangrenous appendices epiploica) (B).

**Figure 3:**
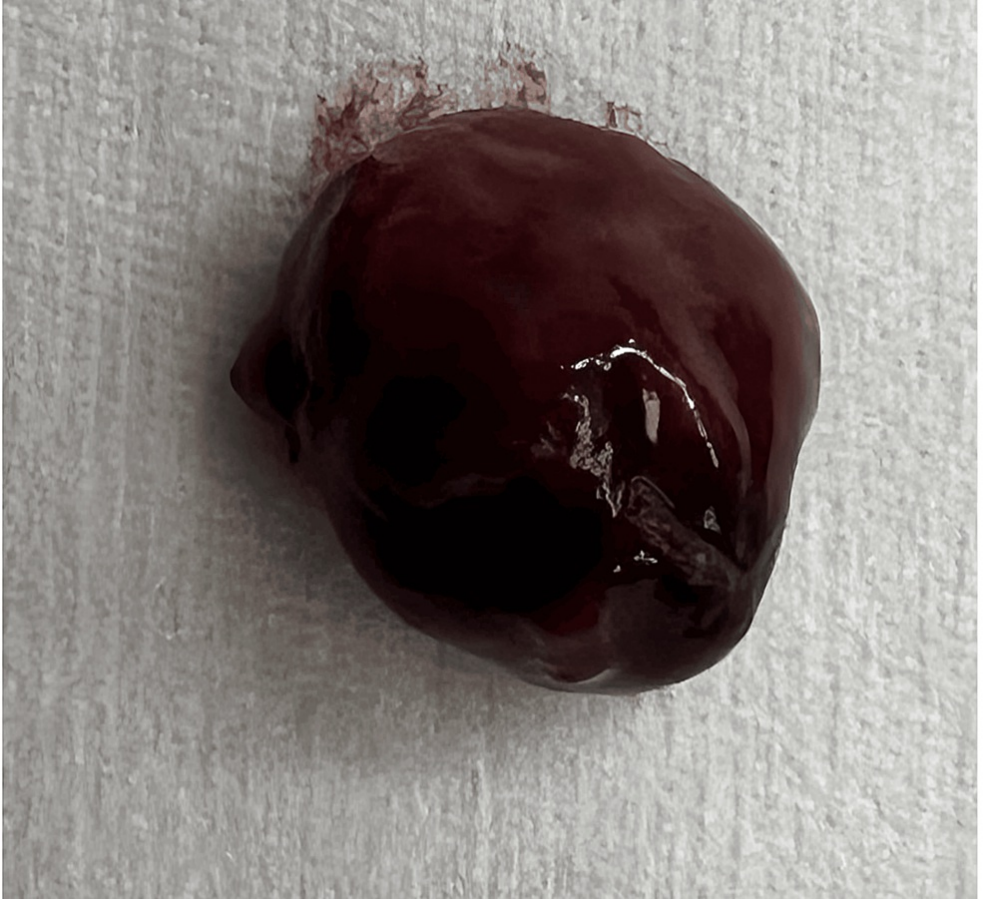
The image shows a resected dark reddish-brown lesion, likely a necrotic epiploic appendage, presented against a light-gray background for contrast.

Given the persistence of symptoms and imaging findings, a diagnostic laparoscopy was performed using the following port placements: a 10 mm umbilical port for the laparoscope, a 5 mm left lower quadrant port 5 cm medial and inferior to the anterior superior iliac spine for the main working instruments, a 5 mm right lower quadrant port mirroring the left for auxiliary instruments, and a 5 mm suprapubic port for retraction and manipulation. Gangrenous appendices epiploica were resected and retrieved through the umbilical port using an endo-bag (Figure 2). A tube drain was placed, and the specimen was sent for histopathological examination (Figure 3). Postoperative management included intravenous fluids, analgesics, and antibiotics (cefepime and metronidazole). The patient's recovery was uneventful, with the resolution of symptoms postoperatively.

## Discussion

Epiploic appendagitis is a rare but significant cause of acute abdominal pain, often misdiagnosed as more sinister conditions, such as diverticulitis, appendicitis, or even colonic tumors. The present case reflects the challenges and complexities involved in diagnosing and managing this condition, echoing the experiences and findings reported in the literature.

The clinical presentation of our patient, characterized by acute severe pain in the left iliac fossa with stable vital signs and negative virology screening, is consistent with previous descriptions of epiploic appendagitis [1]. The absence of systemic infection indicators and the localization of pain were pivotal in narrowing the differential diagnoses. The utility of imaging, particularly CT scans, in diagnosing epiploic appendagitis was crucial in our case, revealing fat stranding and a localized inflammatory process without evidence of other common abdominal pathologies [4]. This finding underscores the value of CT imaging in identifying characteristic features of epiploic appendagitis, such as the "ring sign" or the trapped fat appearance, which are critical in differentiating it from other causes of an acute abdomen [5].

In line with the literature, the management of epiploic appendagitis typically favors conservative approaches, primarily when the diagnosis is clear and complications, such as abscess or significant necrosis, are absent [6]. However, our patient underwent surgical intervention due to the persistence of symptoms and the presence of gangrenous tissue observed during diagnostic laparoscopy, which aligns with the recommendations for surgical management in cases where conservative measures fail or complications develop [7].

Postoperative care in cases of epiploic appendagitis, as seen in our patient, involves supportive measures, such as intravenous fluids, analgesics, and antibiotics. Moreover, postoperative complications in surgeries for epiploic appendagitis, although rare, can include infection, bleeding, and abscess formation at the site of resection. In addition, patients may experience adhesions, which can lead to chronic pain or bowel obstruction. Another possible complication is hernia formation at the port sites. This regimen is consistent with the broader medical literature that suggests a supportive and symptomatic treatment approach post-surgery, ensuring a rapid and uneventful recovery [8].

This case also highlights a rare instance of concurrent omental fat necrosis, which added complexity to the clinical picture and required careful interpretation of imaging studies to ensure accurate diagnosis [9]. The successful resolution of symptoms postoperatively in our patient demonstrates the efficacy of a tailored surgical approach, supported by the comprehensive understanding of the pathology and clinical guidelines provided by prior research.

In conclusion, this case serves as a valuable addition to the existing body of knowledge on epiploic appendagitis, emphasizing the need for high clinical suspicion and the appropriate use of diagnostic imaging to guide management decisions. It also highlights the potential for atypical presentations and the role of surgical intervention in selected cases, thus contributing to the ongoing discourse on optimizing care for patients with this often-overlooked diagnosis [10].

## Conclusions

This case of epiploic appendagitis with concurrent omental fat necrosis highlights the critical need for awareness and precision in diagnosing uncommon causes of acute abdominal pain. Effective use of imaging and timely surgical intervention is essential for the optimal management and prevention of complications, enhancing outcomes in similar clinical scenarios.
